# Boron Nitride Nanoparticles Loaded with a Boron-Based Hybrid as a Promising Drug Carrier System for Alzheimer’s Disease Treatment

**DOI:** 10.3390/ijms23158249

**Published:** 2022-07-26

**Authors:** Özge Çağlar Yıldırım, Mehmet Enes Arslan, Sena Öner, Ivana Cacciatore, Antonio Di Stefano, Adil Mardinoglu, Hasan Turkez

**Affiliations:** 1 Department of Molecular Biology and Genetics, Erzurum Technical University, 25050 Erzurum, Turkey; ozge.caglar@erzurum.edu.tr (Ö.Ç.Y.); enes.aslan@erzurum.edu.tr (M.E.A.); senaoner02@gmail.com (S.Ö.); 2 Department of Pharmacy, University “G. d’Annunzio” of Chieti-Pescara, Via dei Vestini 31, 66100 Chieti Scalo, Italy; ivana.cacciatore@unich.it (I.C.); antonio.distefano@unich.it (A.D.S.); 3 Science for Life Laboratory, KTH-Royal Institute of Technology, SE-17121 Stockholm, Sweden; 4 Centre for Host-Microbiome Interactions, Faculty of Dentistry, Oral & Craniofacial Sciences, King’s College London, London SE1 9RT, UK; 5 Department of Medical Biology, Faculty of Medicine, Atatürk University, 25240 Erzurum, Turkey; hturkez@atauni.edu.tr

**Keywords:** hexagonal boron nitride, folic acid, boron lipoic acid, Alzheimer’s disease, experimental Alzheimer’s disease model

## Abstract

The search for an innovative and effective drug delivery system that can carry and release targeted drugs with enhanced activity to treat Alzheimer’s disease has received much attention in the last decade. In this study, we first designed a boron-based drug delivery system for effective treatment of AD by integrating the folic acid (FA) functional group into hexagonal boron nitride (hBN) nanoparticles (NPs) through an esterification reaction. The hBN-FA drug carrier system was assembled with a new drug candidate and a novel boron-based hybrid containing an antioxidant as BLA, to constitute a self-assembled AD nano transport system. We performed molecular characterization analyses by using UV-vis spectroscopy, Fourier transform infrared spectrophotometer (FTIR), scanning electron microscope (SEM), Energy-dispersive X-ray spectroscopy (EDS) and Zeta potential investigations. Second, we tested the anti-Alzheimer properties of the carrier system on a differentiated neuroblastoma (SHSY5-Y) cell line, which was exposed to beta-amyloid (1–42) peptides to stimulate an experimental in vitro AD model. Next, we performed cytotoxicity analyses of synthesized molecules on the human dermal fibroblast cell line (HDFa) and the experimental AD model. Cytotoxicity analyses showed that even higher concentrations of the carrier system did not enhance the toxicological outcome in HDFa cells. Drug loading analyses reported that uncoated hBN nano conjugate could not load the BLA, whereas the memantine loading capacity of hBN was 84.3%. On the other hand, memantine and the BLA loading capacity of the hBN-FA construct was found to be 95% and 97.5%, respectively. Finally, we investigated the neuroprotective properties of the nano carrier systems in the experimental AD model. According to the results, 25 µg/mL concentrations of hBN-FA+memantine (94% cell viability) and hBN-FA+BLA (99% cell viability) showed ameliorative properties against beta-amyloid (1–42) peptide toxicity (50% cell viability). These results were generated through the use of flow cytometry, acetylcholinesterase (AChE) and antioxidant assays. In conclusion, the developed drug carrier system for AD treatment showed promising potential for further investigations and enlightened neuroprotective capabilities of boron molecules to treat AD and other neurodegenerative diseases. On the other hand, enzyme activity, systematic toxicity analyses, and animal studies should be performed to understand neuroprotective properties of the designed carrier system comprehensively.

## 1. Introduction

In recent years, the incidence of neurodegenerative diseases has increased rapidly with the aging human population. Alzheimer’s disease (AD) ranks first among neurodegenerative diseases, and it is predicted that its incidence will increase by 30% soon [1,2,3]. It is known that the most effective target for clinically used drugs for AD treatment is acetylcholinesterase inhibitors. However, many problems are encountered in delivering these drug candidates to the central nervous system (CNS). Among these complications, bio-metabolization, solubility and difficulties in passing through the brain–blood barrier (BBB) come to the fore. To prevent such obstacles, new drug transport systems should be developed to deliver potential drug candidates to the target region before they are eliminated from circulation [4,5]. Nanoparticles have great potential in overcoming drug delivery problems with various properties such as escaping the immune system, long half-life in circulation and good systemic excretion [6,7].

Boron compounds were investigated in different studies, and it has been shown that various types of boron-based molecules could be promising biomaterials in medical applications with a low cytotoxic profile [8,9,10,11,12]. Moreover, increasing attention to the biological use of hexagonal boron nitride (hBN) nanomaterials for medical applications in different areas such as biosensors, tissue engineering and especially drug delivery put forth favorable features of boron molecules in treatment of neurodegenerative diseases [13,14,15,16]. Boron nitride nanotubes (BNNT) and hBNs are promising nanocarrier systems because of their multipotent features such as excellent physical durability, large surface area and favorable biocompatibility. BNNT and hBN nanoparticles have been studied in various cellular uptake investigations, and it has been confirmed that the hyaluronic acid (HA) and folic acid (FA) conjugations enhance their passage through different environments to reach desired targets [17,18]. Moreover, FA corporation onto BNNT has also been investigated to have a favorable doxorubicin hydrochloride (DOX) loading capacity and exhibit pH-sensitive drug release [19]. In addition, FA integrated BNNT through an esterification reaction can attach, and it can be internalized by cellular systems through FA receptor-mediated endocytosis [20,21].

Self-assembling molecules by non-covalent bonds to constitute a higher ordered structure was shown to build the most potent drug delivery systems. Self-association of molecules led us to construct biomimetic architectures from simpler components to more complex systems to obtain favorable properties [22,23,24,25]. Molecular self-assembly aided the easy and fast production of nanostructures and the construction of advanced multipotent drug carrier molecules. With the development of self-assembling properties of nano-molecules, the advanced biomaterial field enabled the discovery of multifunctional biomaterials, including oligonucleotides, peptides and polysaccharide scaffolds [26,27,28]. It was shown that using self-assembling nano-A6K peptide as a boron molecule carrier had a promising potential in boron neutron capture therapy (BNCT) by simply incubating A6K and sodium borocaptate (BSH) [29]. Moreover, a self-assembling nanomicellar boron transport system was suggested to constitute a thermoresponsive boron-containing poly (N-isopropylacrylamide) diblock copolymer, which can be utilized to build a tumor-selective delivery with significant retention for BNCT [30]. From a different perspective, boron nitride nanoplatelet supported super molecular aggregate (DPCS) was used to construct a self-assembling layer via electrostatic interaction and ionic bonding. This system was shown to have a good flame retardation efficiency with an additional friction supporting property [31].

In this study, a boron-based self-assembling drug transporter system was designed by integrating the BLA into hBN as a nanocarrier to develop a potential anti-Alzheimer’s disease agent with a favorable biocompatibility feature. To construct the self-assembling structure, FA was attached to the hBN surface via the esterification reaction, and the BLA interacted with the transporter through a simple incubation step. Molecular characterizations of the carrier system were performed by using UV-vis spectroscopy, FTIR, SEM, EDS and Zeta potential analyses. The anti-Alzheimer potential of the drug delivery system was explored in the experimental AD model by using cytotoxicity investigations, flow cytometry and acetylcholinesterase (AChE) and antioxidant assays. Moreover, a drug release assay was performed to monitor the efficiency of the self-assembling nano-drug carrier system. Finally, we developed a fast, efficient and biocompatible nano-drug delivery system targeting AD to propose a new treatment strategy for AD and other neurodegenerative diseases. Ultimately, it was aimed to constitute an effective and safe boron-based drug carrier system targeting AD through using self-assembling nano-system machinery.

## 2. Results

**Figure 1:**
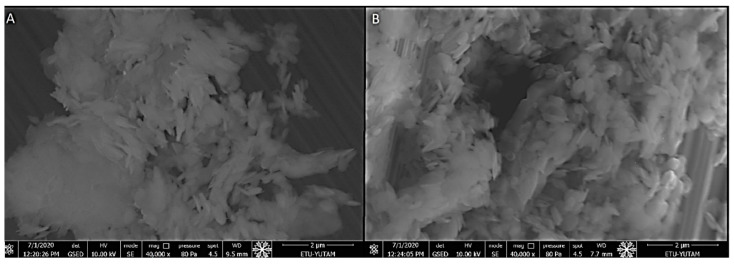
SEM image: (**A**) hBN; and (**B**) hBN-FA.

**Figure 2:**
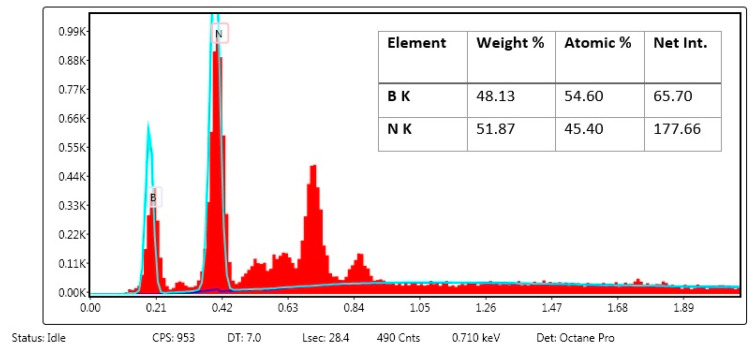
EDX analysis of hBN nanoparticles.

We synthesized hBN nanoparticles by the chemical vapor deposition method. Nanoparticle surface morphology and elemental analysis were performed by EDX-SEM analysis. We observed that the average particle size was around 200–250 nm and had a remarkably homogeneous appearance (Figure 1). EDX analysis shows that the percent atomic content of the hBN nanostructures is composed of the expected atomic ratio (Red lines represent atomic mass and boundary lines represent standard deviations of the data). Moreover, the EDX spectra revealed the presence of B and N atoms (Figure 2).

**Figure 3:**
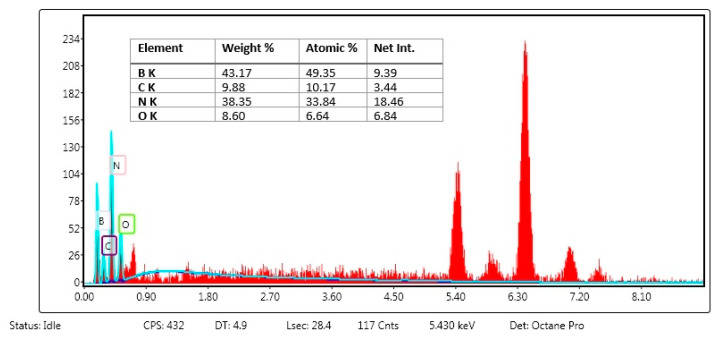
EDX analysis of the hBN-FA complex.

As a result of the esterification reaction between the hydroxyl groups of hBN and the carboxyl groups of FA, we attached FA molecules to the surface of the hBN nanostructures. EDX-SEM micrographs exhibited that the surface morphology of the particle did not change significantly and had a spherical form. The presence of chemical content and percentage of FA was confirmed in the EDX analysis of the hBN-FA nanostructure. Here, the percent ratio of C, H and O atoms in the hBN-FA structure was correlated with expected atomic ratios (Figure 3).

**Figure 4:**
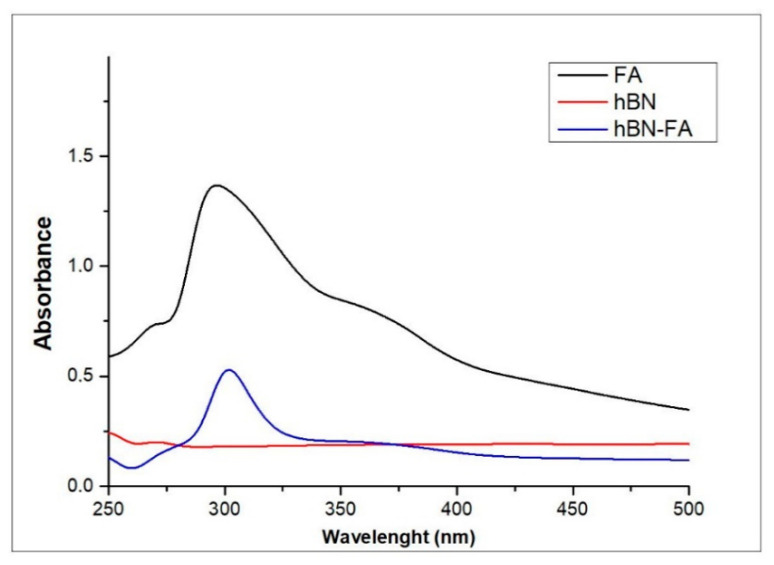
UV-vis spectrum of hBN, FA and hBN-FA.

**Figure 5:**
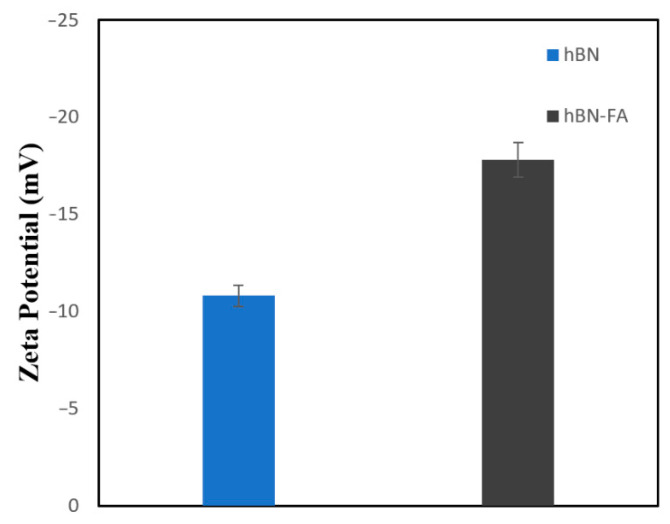
Zeta potentials of hBN and the hBN-FA complex.

FA had a max peak at 290 nm in the UV-vis spectrum, so UV-vis measurement was taken at 290 nm for both hBN and hBN-FA. The peak at 290 nm observed in hBN-FA samples revealed that FA was successfully conjugated to hBN nanoparticles because of the difference in peak shifts (Figure 4). Moreover, Zeta potential analysis supported the successful conjugation of FA. The energy of −10.8 mV of hBN decreased to −17.4 with the integration of FA. In addition, the PDI of hBN and hBN-FA samples were calculated as 0.368 and 0.584, respectively. The Zeta potential analysis (Figure 5) also provided information about the dimension. The calculated average particle size of 260 and 680 nm for hBN and hBN-FA supported SEM imaging.

**Figure 6:**
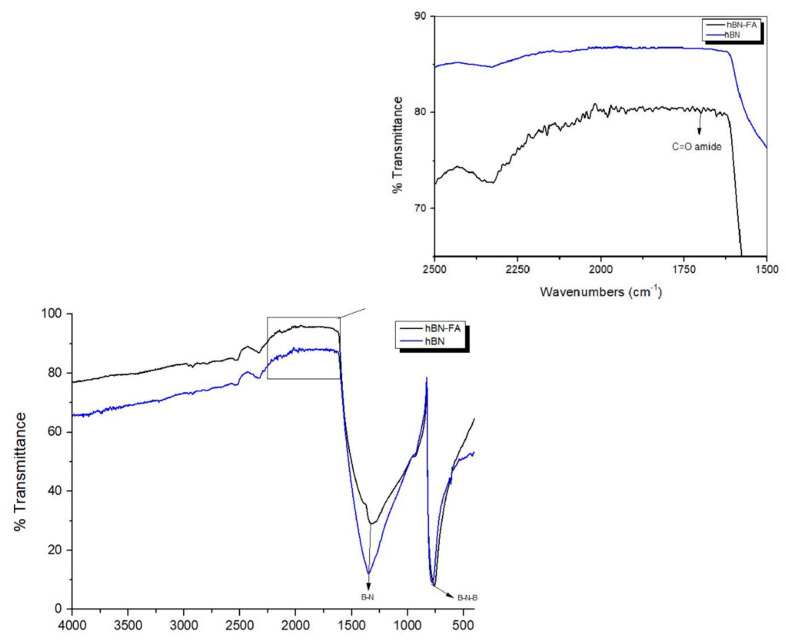
FTIR spectra of hBN and hBN-FA.

In Figure 6, where the FTIR spectrum of the hBN and the hBN-FA complexes is given, the B-N-B vibration of hBN nanoparticles was at 772 cm^−1^, while the stretching vibration of B-N was around 1440 cm^−1^. In addition, the vibration confirming the C=O amide bond was shown at 1707 cm^−1^. In previous studies, B-N-B vibration is given at 780 cm^−1^, B-N at 1540 cm^−1^, and the vibration frequency supporting folic acid is given at C=O amide bond at 1700 cm^−1^ [32,33].

**Figure 7:**
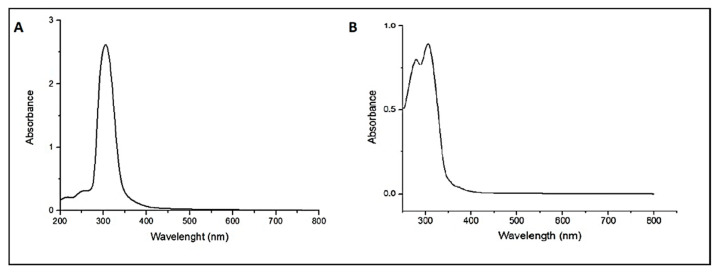
UV-vis spectra of MEM and BLA at the range of 200–800 nm: (**A**) MEM; (**B**) BLA.

We evaluated the loading and release potentials of the MEM and the BLA drugs of hBN and the hBN-FA nanostructures by UV-vis spectroscopy. We determined the maximum peak of both the MEM and the BLA drugs in the 200–800 nm visible region. According to the results presented in Figure 7A,B, the peak values for MEM are 300–310 nm. Two loading experiments, 1:1 and 1:2, of the MEM and the BLA drugs were carried out on hBN nanoparticles. The hBN nanoparticles showed 40.1% and 84.3% drug-loading capacity for MEM drug at 1:1 and 1:2 loading ratios, respectively. The nanoparticle did not load the BLA drug at either mixing ratio. On the other hand, the hBN-FA nano conjugate loaded the BLA drug 97.5% and 99.8% for 1:1 and 1:2 ratios, respectively. FA conjugated hBN nanoparticles also exhibited an increased drug-loading capacity for 1:1 and 1:2 ratios of MEM as 52.6% and 95%, respectively (Table 1).

**Figure 8:**
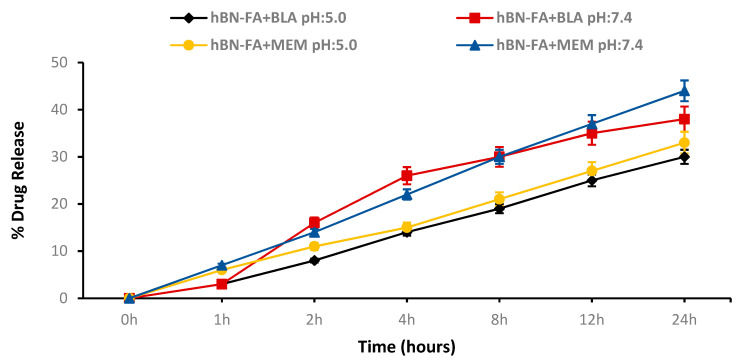
Drug release study for pH:5.0 and pH: 7.4.

Release experiments of the MEM and the BLA drugs loaded on the hBN-FA nanocarrier were carried out in two different PBS environments, pH: 5.0 and pH: 7.4. We observed that the drug release value was calculated by using the samples taken from the environment at certain time intervals (0-1-2-4-8-12-24 h) increased in all groups depending on the time. The results showed that the hBN-FA nanocarrier exhibited a compelling time-dependent release profile for the MEM and the BLA drugs. After 24-h observations, the percentage of the release of the hBN-FA nanocarrier MEM and BLA drugs was calculated as 33% and 30%, respectively, at pH: 5.0, while release values were 44% and 38% at pH: 7.4 (Figure 8).

**Figure 9:**
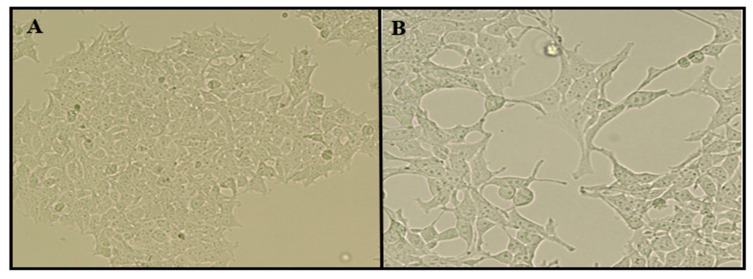
SHSY-5Y cells are differentiated into neuron-like cells by retinoic acid treatment: (**A**), negative control; and (**B**), 10 µM of all trans RA applications for 11 days.

SHSY-5Y neuroblastoma cell line was differentiated into a mature neuron cell-like culture after 11 days of retinoic acid treatment. After the differentiation procedure, cell cultures were investigated by using an inverted microscope, and we observed that cell bodies were turned into thinner and more elongated structures from cubic and ellipsoid cellular shapes. Moreover, elongated axon and dendrite-like structures are formed, and cellular interactions became visible between each cell (Figure 9). Moreover, flow cytometric cell cycle analysis showed that the retinoic acid applied cell culture population shifted from the S and the G2 cell cycle phase into the G1 phase. These results showed that the cell cultures were transformed from DNA synthesis into interphase, similar to neuronal cells (Table 2).

**Figure 10:**
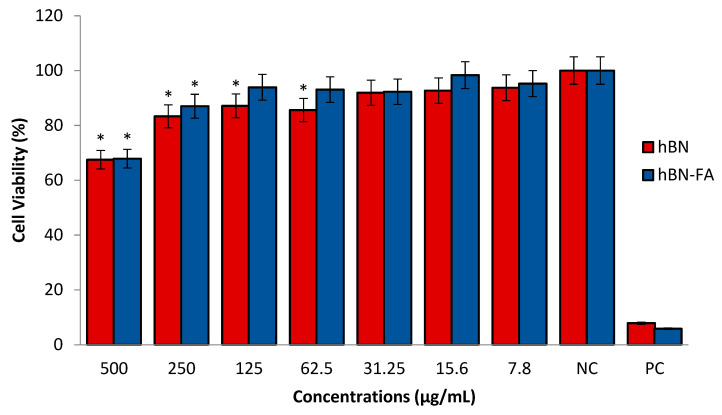
In vitro cytotoxicity of hBN and hBN-FA on human dermal fibroblast cells (HDFa) for 24 h. Symbol (*) represents a statistically significant difference compared to the negative control.

**Figure 11:**
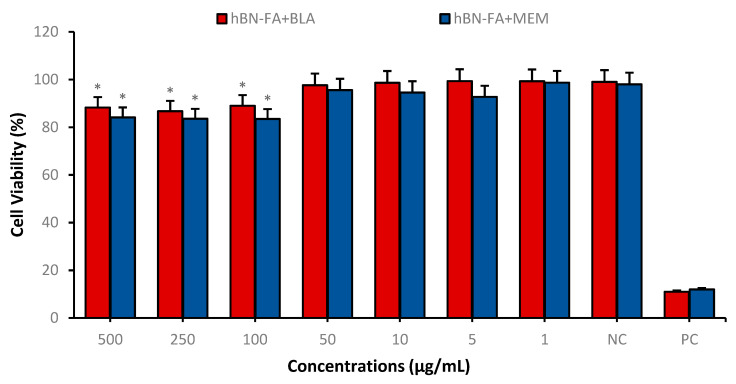
Cytotoxicity of hBN-FA+BLA and hBN-FA+MEM (0–500 µg/mL) on differentiated SHSY-5Y cell lines for 24 h using MTT assay. Symbol (*) represents a statistically significant difference compared to the negative control.

We performed cytotoxicity analyses of hBN and the carrier system (hBN-FA) in a healthy human fibroblast cell line (HDFa) to assess the toxicological properties of unloaded hBN-FA. We observed that over 62.5 µg/mL concentration of hBN had significant toxicity on the HDFa cell line. Moreover, 250 µg/mL concentration of hBN-FA showed significant cytotoxicity in the HDFa cell culture compared to the negative control (Figure 10). After the differentiation procedure, the synthesized hBN-FA carrier system was loaded with boron lipoic acid (BLA) and memantine (MEM) molecules. Drug loading was performed by mixing hBN-FA and BLA or MEM to constitute a self-assembling drug interaction. Cytotoxicity properties of hBN-FA+BLA and hBN-FA+MEM (0–500 µg/mL) were assessed on differentiated SHSY-5Y cell lines for 24 h of applications by using an MTT cell viability assay. Cytotoxicity analysis showed that both BLA and MEM-loaded hBN-FA have significant cytotoxicity on the differentiated cell cultures at 500, 250 and 100 µg/mL concentrations. On the other hand, there was no significant cytotoxic effect of hBN-FA+BLA and hBN-FA+MEM on cell cultures under 50 µg/mL concentration (Figure 11).

**Figure 12:**
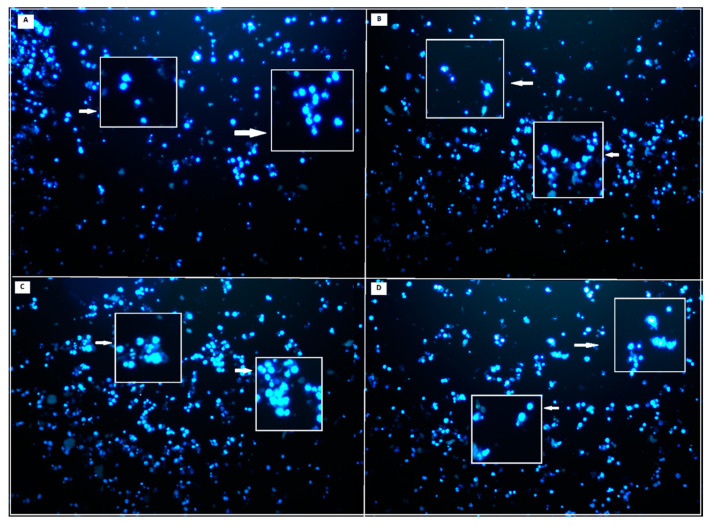
Hoechst 33258 fluorescent staining of differentiated SHSY-5Y cell lines against 25 µg/mL concentration of hBN-FA+BLA, hBN-FA+MEM and hBN-FA applications for nuclear abnormalities for 24 h (20× magnifications): (**A**) hBN-FA+BLA; (**B**) hBN-FA+MEM; (**C**) only β-amyloid (**D**) hBN-FA.

Genotoxic properties of hBN-FA+BLA, hBN-FA+MEM and hBN-FA were analyzed by using the Hoechst 33258 fluorescent nuclear staining technique and white arrows indicate abnormal nuclear structure (white box shows 4× magnified sections). According to the investigations, there was no significant difference between the negative control group and hBN-FA+BLA, hBN-FA+MEM and hBN-FA applications. On the other hand, the β-amyloid application was found to significantly increase the nuclear abnormality compared to the negative control (Figure 12 and Table 3).

**Figure 13:**
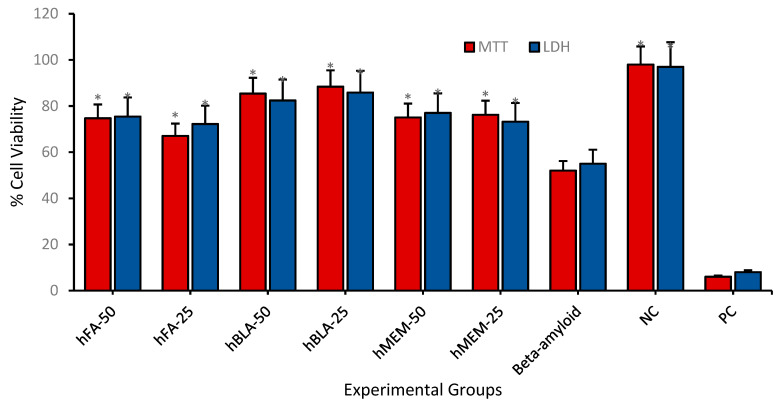
Cell viability tests (MTT and LDH assays) hBN-FA, hBN-FA+BLA, hBN-FA+MEM and β-amyloid (50 µg/mL and 25 µg/mL) on the experimental AD model for 24 h. Symbol (*) represents significantly increased cell viability (*p* < 0.0001) compared to only beta-amyloid applied groups.

**Figure 14:**
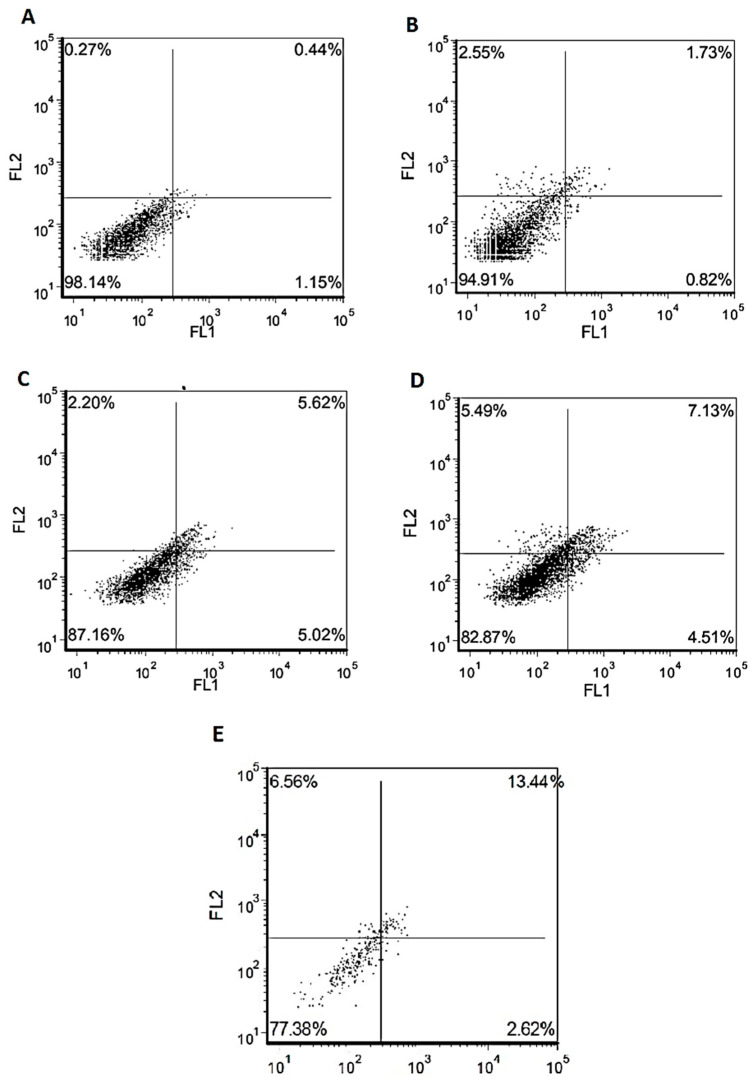
Flow cytometry analysis (annexin V- (FL1-H) and PI- (FL2-H) labeled cells) of hBN-FA+BLA, hBN-FA+MEM, hBN (25 µg/mL) against beta-amyloid cytotoxicity: (**A**) negative control; (**B**) hBN-FA+BLA; (**C**) hBN-FA+MEM; (**D**) hBN-FA; (**E**) only β-amyloid. Statistical analysis was performed using a one-way ANOVA followed by Tukey’s post hoc test.

After cytotoxicity investigations on the differentiated cell cultures, we analyzed nontoxic concentrations of hBN-FA+BLA and hBN-FA+MEM formulations in the experimental AD model, where the disease toxicity was stimulated by beta-amyloid 1–42 exposure. When the carrier system (hBN-FA) was applied to the experimental AD model at concentrations of 50 and 25 µg/mL, there was a significant cell viability increase compared to the beta-amyloid used group. Moreover, MEM-loaded hBN-FA showed higher cell viabilities at both concentrations compared to the hBN-FA applied AD model. In addition, the highest cell viability ratios were obtained from 25 and 50 µg/mL of hBN-FA+BLA with 88% and 77% living cells, respectively, in the experimental AD model (Figure 13). Flow cytometry analysis showed that 25 µg/mL of hBN-FA+BLA exposure on the AD model increased cell viability by 20% compared to the only beta-amyloid applied cell cultures. Moreover, 25 µg/mL of hBN-FA+MEM application alleviated cytotoxicity by about 10% in on the AD model. On the other hand, 25 µg/mL of hBN-FA application only increased cell viability about 9% ratio compared to the amyloid beta applied cell cultures. Our results indicated that BLA-loaded hBN-FA has a higher ameliorative effect on the experimental AD model compared to the hBN-FA+MEM application (Figure 14).

**Figure 15:**
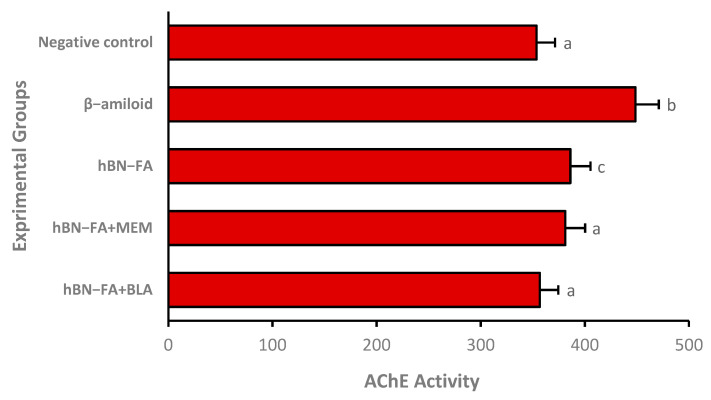
The effects of hBN-FA+BLA, hBN-FA+MEM, and hBN (25µg/mL) applications on Aβ1-42-induced AChE activity. (Each superscript letter (^a, b, c^) represents statistically similar groups).

Acetylcholinesterase (AChE) enzyme activity is one of the most critical parameters used in the development of drugs for AD treatment. We investigated AChE activities after the drug carrier systems were applied to the AD model by loading BLA and MEM. A total of 25 µg/mL of hBN-FA+MEM and hBN-FA+BLA were used in the experimental AD model for 24 h, and AChE activities were analyzed at the end of the incubation period. We observed that both hBN-FA+MEM and hBN-FA+BLA application alleviated the AChE activity, and the activity levels were found very similar to the negative control group, differentiated cell culture without beta-amyloid application. Although hBN-FA application decreased the AChE enzyme activity, it was not alleviated to the negative control AChE activity levels (Figure 15). We also investigated the antioxidant and oxidative stress parameters in the experimental AD model. According to the results, the BLA-loaded hBN-FA carrier system ameliorated oxidative stress resulting from beta-amyloid application and significantly increased antioxidant status compared to only the beta-amyloid applied group. On the other hand, the MEM-loaded carrier system could only balance the oxidative status, but it could not increase antioxidant status to reach negative control levels (Table 4).

## 3. Discussion

For more than 20 years, researchers have been conducting revolutionary studies in various applications by using the unique properties of nano-sized materials. Nanotechnology allows the development of many technologies in diverse industries such as information technology, security, transportation, medicine, food, environment, space research, electronics, communication and the defense industry [34]. Due to their unique properties, such as optics, magnetic or electronics at the nanoscale, nanomaterials also attract great attention in biomedicine [35,36]. Thus, nanomaterials exhibit unique properties compared to bulk materials, and they can be designed to interact with cells or tissues at the molecular level for their applications in medicine. Nanotechnology is not a stand-alone scientific discipline but consists of interactions from different traditional sciences such as chemistry, biology, materials science and physics [37]. Nanotechnology holds the multifaceted promise of improving existing techniques and enabling entirely new scientific developments to emerge. By manipulating drugs and other materials at a nanometer scale, materials’ essential properties or bioactivity can be changed. Thus, many factors such as solubility or retention time in the bloodstream, controlled release for short or long periods and target site-specific release can be manipulated [38]. In this study, we have designed a boron-based drug delivery system by integrating an FA functional group into hBN NPs through an esterification reaction. To constitute a self-assembled drug carrier system, we combined hBN-FA with the BLA compound, a new drug candidate for targeting AD pathophysiology.

First, hBN nanoparticles were synthesized by the chemical vapor deposition method and FA was covalently attached to the surface of hBN nanoparticles by the esterification reaction. In previous studies, an hBN thin film was successfully synthesized on different substrates such as silicon and sapphire by low-pressure chemical vapor deposition [39,40]. Moreover, it was shown that the functionalization of BN by incorporating with FA has an excellent advantage as a drug delivery system [41,42]. A previous study showed that folate-conjugated boron nitride nanotubes can be used as a boron carrier for boron neutron capture therapy (BNCT) in cerebral glioblastoma multiforme. Moreover, it was shown that the folate group can be used as a selective tumor targeting ligand for drug delivery systems [43]. In addition, folic acid functionalized boron nitride oxide was shown in a computational biology study to be a carrier molecule that has great potential for cancer drug delivery. In the study, the ludarabine (Flu) and cytarabine (Cyt) cancer drug-loading capacity of the drug delivery system was calculated by using computational simulations, and the results showed that the folic acid conjugated boron nitride oxide yielded promising result for cancer treatments [44]. Synthesized compounds were characterized and confirmed by SEM and EDX analyses. Integration of FA onto hBN NPs was investigated by UV-Vis spectrophotometer at 290 nm and zeta potential analyses confirmed the incorporation.

Moreover, molecular size analyses showed a synthesized drug delivery system at a nano level around 600 nm. Furthermore, FTIR analyses showed a difference in peak formation between hBN and hBN-FA, confirming the FA integration onto hBN molecules. Studies have reported that drug/gene nanocarrier systems prepared using FA ligands exhibit low toxicity on healthy tissues but had effective results in treatment of tumors due to more specific cellular uptake. The influence of FA’s small size, low immunogenicity and unique pharmacokinetic properties makes it a suitable candidate for functionalizing drug carrier systems [45]. Moreover, drug-loading investigations showed that hBN NPs could load memantine significantly. In addition, hBN NPs did not show any loading capacity against the BLA molecules. On the other hand, the hBN-FA drug carrier system showed greater drug-loading capacity for BLA than memantine. This loading capacity change probably resulted from charge reversal from FA integration into hBN NPs. Several studies reported that charge reversal modifications significantly increase the carrier molecule’s drug-loading capacity [45,46]. Moreover, drug release assays showed that the hBN-FA drug carrier system exhibited higher drug release capacity for the phycological condition at pH:7.4, giving greater potential for the drug carrier applicability.

A healthy HDFa cell line and differentiated SH-SY5Y cell culture were used to assess cytotoxicity, genotoxicity and anti-AD potential of the carrier system and the drug-loaded carriers. According to the toxicity analyses on the HDFa cell line, we found that FA integration into hBN decreased cytotoxicity significantly compared to hBN. In addition, the cytotoxic concentration of hBN increased from 62.5 µg/mL to 250 µg/mL after FA incorporation. In agreement with our results, previous studies investigated that functionalization by FA integration could significantly reduce drug transport molecules’ cytotoxicity [47,48]. Next, we analyzed the cytotoxicity analyses of drug-loaded hBN-FA on the differentiated SH-SY5Y cell cultures and defined the non-toxic dose intervals for anti-AD studies. We observed that both MEM and BLA-loaded hBN-FA carriers did not show cytotoxicity under 50 µg/mL concentration for the differentiated SH-SY5Y cell cultures. Furthermore, genotoxicity investigations showed no significant difference in nuclear abnormality after hBN-FA+BLA, hBN-FA+MEM and hBN-FA applications at different concentrations compared to the negative control.

Finally, the experimental AD model was constituted from a beta-amyloid application to the differentiated SH-SY5Y cell culture. Non-toxic concentrations of hBN-FA and drug-loaded hBN-FA (50 µg/mL and 25 µg/mL) were analyzed in the experimental AD model. As a result of these experiments, we found that BLA-loaded hBN-FA has the highest potential in neuroprotection compared to the MEM-loaded carrier system. A total of 25 µg/mL concentration of BLA-loaded carrier exhibited a 33% increase in cell viability compared to only beta-amyloid applied cell cultures. These results were generated based on cell viability assays and flow cytometry analyses. An increase in the AChE activity is one of the essential pathologies of AD. We found that the enzyme activity was increased to lead to neuronal cell deaths by enhancing senile plaque formations [49,50]. Moreover, AChE inhibitors are currently utilized for AD patients, and the discovery of AChE inhibitors is still a hot topic in the treatment of AD and other neurodegenerative diseases [51,52]. Furthermore, oxidative stress was shown to have a close relationship with AD progression, which was led by mitochondrial dysfunction and enhancement of neurodegeneration [53,54]. In our study, we found that the BLA-loaded drug carrier system could significantly increase antioxidant levels and significantly decrease the oxidative status in the experimental AD model compared to the beta amyloid-treated group.

## 4. Materials and Methods

### 4.1. Preparation of hBN-FA Nano Conjugates

The hBN nanoparticles were prepared by the chemical vapor deposition method [55]. Folic acid (FA) was covalently attached to the surface of hBN nanoparticles by the esterification reaction. Briefly, 10 mg of hBN was treated with 10 mL H_2_0_2_ for 1 h and stirred overnight at 70 °C. The aim of this process is to add -OH group onto the hBN surface as an active site for further reactions. The hBN-OH was obtained from the reaction mixture by centrifuging at 9000 rpm for 10 min and washed with dH_2_O twice. A total of 10 mg FA, 10 mg N-(3-dimethylaminopropyl)-N’-ethylcarbodiimide hydrochloride (EDC) and 6.7 mg 4-(dimethylamino)-pyridine (DMAP) were suspended simultaneously in 20 mL N, N-dimethylacetamide (99.8%) then the sample mixed for 30 min. Then 10 mg of dried hBN-OH was added to the mixture and stirred overnight at room temperature. The hBN-FA nanoconjugates were collected by centrifugation at 9000 rpm for 15 min. The hBN-FA nanostructures were suspended in PBS for particle characterization study.

### 4.2. Characterization

The surface morphology of hBN and hBN-FA nanoparticles was investigated with a scanning electron microscope (Quanta FEG 250, FEI, Eindhoven, The Netherlands), and chemical characterization of nanoparticles was performed via the use of energy-dispersive X-ray spectroscopy (EDS, EDX). Particle size and polydispersity index (PDI) were measured by using Zeta Sizer (ZS Nano, Malvern Panalytical, Malvern, UK). Fourier transform infrared (FTIR) spectrum was performed using Bruker VERTEX 70v in the 400–4000 cm^−1^ range. Ultraviolet-visible absorption spectra (UV-vis) of hBN, hBN-FA and FA were performed in the 200–800 nm range.

### 4.3. Human Dermal Fibroblast (HDFa) Cell Culture

Human dermal fibroblast cell lines (HDFa) were grown in DMEM medium including fetal bovine serum (FBS) 10% and supplemented 100 U/mL penicillin-streptomycin antibiotic. Culture dishes were kept in a culture incubator at 37 °C with 5% CO_2_ until they reached a defined density (80% confluency). Cells washed with PBS were treated with trypsin-EDTA for 5 min 37 °C. Then the samples were centrifuged for 5 min at 3000 rpm. The pellet was dissolved with fresh DMEM medium.

### 4.4. 3-(4,5-Dimethylthiazol-2-yl)-2,5-diphenyl-2H-tetrazolium Bromide (MTT) Assay

The MTT method was performed as described in a previous study [56]. Briefly, HDFa were counted using a hemacytometer. Cells were seeded as 2 × 10^4^ cells per well in a 48-well plate and the plate incubated with different concentrations of hBN and hBN-FA (0–500 µg/mL) at 37 °C in humidified 5% CO_2_ incubator for 24 h. At the end of the incubation period, 10 µL of MTT reagent was added per well, and the plate was kept in the culture incubator for 3 h. After the incubation period, formazan crystals were dissolved in DMSO (Sigma-Aldrich^®^, St. Louis, MO, USA), yielding purple color in the cultures. Samples were analyzed using a microplate reader at a wavelength of 570 nm.

### 4.5. Drug Loading and Release

Drug-loading analyses were performed by mixing 2 mL of hBN and hBN-FA complex (0.5 mg/mL in PBS) with 100 μL (1 mg/mL) of boron lipoic acid (BLA) and 100μL (0.5 mg/mL) of memantine (MEM) as described in a previous study [33] with minor modifications. The carrier systems and drugs were mixed for drug-loading analyses at room temperature in a dark place for 24 h. After this step, the concentrations of drugs in the supernatant were determined by using the Nanodrop 2000 spectrophotometer.

For the drug release test, 1 mg of hBN+ MEM/BLA complex were suspended in 2 mL of PBS (pH 7.4 and 5.0), and the sample was mixed at room temperature on a vertical shaker. Then 0.5 mL of PBS was removed from the medium, and the same volume of fresh PBS was added to the suspension in certain time intervals (1 to 24 h in 2 h intervals). The drug release quantities were measured by Nanodrop spectrophotometer for hBN-FA+MEM and hBN-FA+BLA.

### 4.6. SHSY-5Y Cell Culture and In Vitro Alzheimer Disease (AD) Model

SHSY-5Y (ATCC CRL-2266) cells were seeded in DMEM-F12 medium supplemented FBS 10% and 100 U/mL pen-strep in cell culture flash. The plate was placed in a cell culture incubator at 37 °C with humidified 5% CO_2_. When cells reached 80% confluence, 10 µM all-trans retinoic acid (all-trans-RA) was added to cultures, and cells were incubated for 11 days at 5% CO_2_ at 37 °C (in a low FBS medium). Then morphological changes of cells were monitored under an inverted microscope [56]. Moreover, flow cytometry (CyFlow Cube 6) cell cycle analysis was performed to determinate differentiated cells.

### 4.7. Efficiency of the Drug Delivery System on In Vitro AD Model

The differentiated cell culture was treated with various concentrations of hBN-FA+BLA and hBN-FA+MEM (1, 5, 10, 25, 50, 100, 250, 500 μM) and Aβ1–42 (20 μM) (Sigma-Aldrich^®^, St. Louis, MO, USA) for 24 h (n = 4). A non-treated differentiated SHSY-5Y cell culture was used as the negative control group. The efficiency of the transport system in AD was investigated using MTT, AChE activity assays, flow cytometry analysis, Hoechst nuclear staining and TAC-TOS parameters.

### 4.8. Cell Viability Assay

The differentiated cell culture was treated to different concentrations of hBN-FA+BLA and hBN-FA+MEM (1 to 500 μM) and Aβ1–42 (20 μM) (Sigma-Aldrich^®^, St. Louis, MO, USA) for 24 h. The culture plate was kept in the culture incubator at 37 °C with 5% CO_2_ for 24 h. At the end of the incubation period, the culture medium was removed from the cell plate. Then 10 µL of MTT reagent was added per well, and the plate was kept in the culture incubator for 3 h. After the incubation period, formazan crystals were dissolved in DMSO (Sigma-Aldrich^®^, St. Louis, MO, USA), and the cell culture plate was analyzed using a microplate reader at a wavelength of 570 nm. The biologically effective dose was determined for both hBN-FA+BLA and hBN-FA+ MEM for further analysis.

### 4.9. Flow Cytometry Analyses

Cell cultures were collected from the plate surface by using trypsin after compound applicationsl 10^4^ cells were collected by centrifugation, and cells were resuspended in 500 µL of 1× binding buffer. Next, 5 µL Annexin V-FITC and 5 µL propidium iodide (PI 50 µg/mL) was added to the cultures. Cultures were incubated for 5 min in a dark place. Then, cells were examined via the flow cytometry instrument using a double filter set for FITC and rhodamine.

### 4.10. Acetylcholinesterase (AChE) Activity

Acetylcholinesterase activity was performed according to instructions of the Acetyl Cholinesterase Assay kit (Abcam^®^. Cambridge, MA, USA). A lysis buffer was used to lyse the cells to analyze enzyme activity. Then 50 µL of acetylcholine reaction mix reagent was added to the samples. The plate was kept in the dark for 30 min. The absorbance was measured by the microplate reader at OD = 410 ± 5 nm.

### 4.11. TAC and TOS Analysis

TAC and TOS assays were performed according to the manufacturer’s (Rel Assay Diagnostics^®^, Gaziantep, Turkey) recommendations. Antioxidants were reduced ABST radicals into colorless molecules. The culture supernatant was mixed with Reagent 1 and dH_2_O in 48-well plate. Then, Reagent 2 was added into the 48-well plate. The total antioxidant capacity was calculated at 660 nm for all samples. Oxidant oxides were added to the ferrous ion complex into the ferrous ion in the sample. For TOS analysis, the sample medium was mixed as Reagent 1 and Reagent 2. The absorbance was assessment at 530 nm in a plate reader.

### 4.12. Hoechst 33258 Staining

The differentiated cell culture was treated to different concentrations of hBN-FA+BLA and hBN-FA+MEM (25 μM) and Aβ1–42 (20 μM) (Sigma-Aldrich^®^, St. Louis, MO, USA) for 24 h. At the end of the period, the culture medium was discarded, and cells was washed with PBS. Then the cells were fixed with 4% paraformaldehyde in PBS at 4 °C for 30 min. The samples were incubated with 1 µM Hoechst 33258 fluorescent dye for 5 min at dark and room temperature. The cells were observed under a fluorescent microscope

## 5. Conclusions

In this study, a self-assembling boron-based nano-carrier system was developed for targeting AD. In this context, we synthesized hBN NPs and integrated FA onto NPs by esterification reaction to increase its loading affinity. We observed that the BLA molecule has greater affinity for FA-linked hBN, and it could be hypothesized that FA integration might result in a charge reversal on the hBN NPs. Moreover, we observed that the hBN-FA drug carrier system had an advantage in slow drug release capacity of the BLA molecule. Investigations of anti-AD potential of the BLA-loaded hBN-FA carrier on the experimental AD model showed that BLA exhibited greater neuroprotective properties in the cell culture models compared to the MEM-loaded drug delivery system. Moreover, the drug-loaded carrier system had significant AChE inhibitory effect and antioxidant properties in the experimental AD model.

## Figures and Tables

**Table 1:** Drug loading capacities of carrier system for BLA and Memantine molecules.

Formulations	Ratio	(%) Drug Loading
hBN+MEM	1:2	84.3
hBN+MEM	1:1	40.1
hBN-FA+Memantine	1:2	95
hBN-FA+Memantine	1:1	52.6
hBN+BLA	1:2	-
hBN-BLA	1:1	-
hBN-FA+BLA	1:2	97.5
hBN-FA+BLA	1:1	99.8

**Table 2:** Flow cytometry, cell cycle analyses of differentiated SHSY-5Y cell line into mature neuron cell culture.

	Cell Population (%)			
Group	G1 Phase	G2 Phase	S Phase	G2/G1
Control	34.49 ± 1.72	16.22 ± 0.81	46.27 ± 1.31	3.02 ± 0.15
RA treated	70.96 ± 3.54 *	9.83 ± 0.61 *	15.79 ± 0.78 *	3.42 ± 0.18 *

Values are expressed as the mean ± standard deviation. Symbol (*) represents a statistically significant difference (*p* < 0.01) compared with control.

**Table 3:** Hoechst 33258 fluorescent staining of differentiated SHSY-5Y cell lines hBN-FA+BLA, hBN-FA+MEM and hBN applications for nuclear abnormalities for 24 h. (Each superscript letter (^a, b, c^) represents statistically similar groups).

	Nuclear Abnormalities (NA)			
Groups and Doses	Total MN	Total Lobbed	Total Notched	Mean NA/1000 Cells ± SD
Negative Control	4	3	4	0.011 ± 0.002 ^a^
hBN-FA+BLA (25 µg/mL)	4	3	3	0.010 ± 0.004 ^a^
hBN-FA+BLA (50 µg/mL)	3	3	4	0.010 ± 0.008 ^a^
hBN-FA+MEM (25 µg/mL)	5	4	2	0.011 ± 0.007 ^a^
hBN-FA+MEM (50 µg/mL)	4	5	3	0.012 ± 0.009 ^a^
hBN-FA (25 µg/mL)	5	3	4	0.012±0.003 ^a^
hBN-FA (50 µg/mL)	6	2	2	0.010 ± 0.003 ^a^
β-amyloid (25 µg/mL)	5	6	4	0.015 ± 0.005 ^b^
β-amyloid (50 µg/mL)	9	5	4	0.019 ± 0.001 ^c^

**Table 4:** TAC and TOS values in experimental AD model against hBN-FA+BLA, hBN-FA+MEM and hBN (25 µg/mL) applications for 24 h. (Each superscript letter (^a, b, c, d^) represents statistically similar groups).

Experimental Groups	TAC (mmol Trolox Equiv./L)	TOS (µmol H_2_O_2_ Equiv./L)
Negative Control	1.74 ^a^	0.38 ^c^
hBN-FA+BLA	1.62 ^a^	0.44 ^c^
hBN-FA+MEM	1.13 ^b^	0.87 ^d^
hBN-FA	1.04 ^b^	0.54 ^c^
Only β-amyloid	0.80 ^b^	0.89 ^d^

## Data Availability

The data presented in this study are available on request from the corresponding author. The data are not publicly available due to privacy.

## References

[1] Karthivashan G., Ganesan P., Park S.-Y., Kim J.-S., Choi D.-K. (2018). Therapeutic strategies and nano-drug delivery applications in management of ageing Alzheimer’s disease. Drug Deliv..

[2] Weller J., Budson A. (2018). Current understanding of Alzheimer’s disease diagnosis and treatment. F1000Research.

[3] Vaz M., Silvestre S. (2020). Alzheimer’s disease: Recent treatment strategies. Eur. J. Pharmacol..

[4] Adnet T., Groo A.-C., Picard C., Davis A., Corvaisier S., Since M., Bounoure F., Rochais C., Le Pluart L., Dallemagne P. (2020). Pharmacotechnical Development of a Nasal Drug Delivery Composite Nanosystem Intended for Alzheimer’s Disease Treatment. Pharmaceutics.

[5] Pardridge W.M. (2020). Treatment of Alzheimer’s Disease and Blood–Brain Barrier Drug Delivery. Pharmaceuticals.

[6] Chao X., Zhao L., Ma N., Mou Y., Zhang P. (2021). Nanotechnology-based drug delivery systems for the improved sensitization of tamoxifen. J. Drug Deliv. Sci. Technol..

[7] Suri S.S., Fenniri H., Singh B. (2007). Nanotechnology-based drug delivery systems. J. Occup. Med. Toxicol..

[8] Türkez H., Arslan M.E., Sönmez E., Geyikoğlu F., Açıkyıldız M., Tatar A. (2019). Microarray assisted toxicological investigations of boron carbide nanoparticles on human primary alveolar epithelial cells. Chem. Biol. Interact..

[9] Turkez H., Arslan M.E., Tatar A., Mardinoglu A. (2021). Promising potential of boron compounds against Glioblastoma: In Vitro antioxidant, anti-inflammatory and anticancer studies. Neurochem. Int..

[10] Türkez H., Arslan M.E., Sönmez E., Tatar A., Açikyildiz M., Geyikoğlu F. (2017). Toxicogenomic responses of human alveolar epithelial cells to tungsten boride nanoparticles. Chem. Biol. Interact..

[11] Turkez H., Cacciatore I., Arslan M.E., Fornasari E., Marinelli L., Di Stefano A., Mardinoglu A. (2020). Histidyl-Proline Diketopiperazine Isomers as Multipotent Anti-Alzheimer Drug Candidates. Biomolecules.

[12] Enes M., Erzurum A. (2016). Ameliorative effect of boric acid against nicotine-induced cytotoxicity on cultured human primary alveolar epithelial cells. J. Boron.

[13] Emanet M., Şen Ö., Çulha M. (2017). Evaluation of boron nitride nanotubes and hexagonal boron nitrides as nanocarriers for cancer drugs. Nanomedicine.

[14] Lu F., Wang F., Cao L., Kong C.Y., Huang X. (2012). Hexagonal Boron Nitride Nanomaterials: Advances Towards Bioapplications. Nanosci. Nanotechnol. Lett..

[15] Sharker S.M. (2019). Hexagonal Boron Nitrides (White Graphene): A Promising Method for Cancer Drug Delivery. Int. J. Nanomed..

[16] Küçükdoğru R., Türkez H., Arslan M.E., Tozlu Ö.Ö., Sönmez E., Mardinoğlu A., Cacciatore I., Di Stefano A. (2020). Neuroprotective effects of boron nitride nanoparticles in the experimental Parkinson’s disease model against MPP+ induced apoptosis. Metab. Brain Dis..

[17] Sharker S.M., Alam M.A., Shill M.C., Rahman G.M.S., Reza H.M. (2017). Functionalized hBN as targeted photothermal chemotherapy for complete eradication of cancer cells. Int. J. Pharm..

[18] Weng Q., Wang B., Wang X., Hanagata N., Li X., Liu D., Wang X., Jiang X., Bando Y., Golberg D. (2014). Highly Water-Soluble, Porous, and Biocompatible Boron Nitrides for Anticancer Drug Delivery. ACS Nano.

[19] Sukhorukova I.V., Zhitnyak I.Y., Kovalskii A.M., Matveev A.T., Lebedev O.I., Li X., Gloushankova N.A., Golberg D., Shtansky D.V. (2015). Boron Nitride Nanoparticles with a Petal-Like Surface as Anticancer Drug-Delivery Systems. ACS Appl. Mater. Interfaces.

[20] Qu W., Meng B., Yu Y., Wang S. (2018). Folic acid-conjugated mesoporous silica nanoparticles for enhanced therapeutic efficacy of topotecan in retina cancers. Int. J. Nanomed..

[21] Kunjiappan S., Pavadai P., Vellaichamy S., Ram Kumar Pandian S., Ravishankar V., Palanisamy P., Govindaraj S., Srinivasan G., Premanand A., Sankaranarayanan M. (2021). Surface receptor-mediated targeted drug delivery systems for enhanced cancer treatment: A state-of-the-art review. Drug Dev. Res..

[22] Ruiz-Hitzky E., Darder M., Aranda P., Ariga K. (2010). Advances in Biomimetic and Nanostructured Biohybrid Materials. Adv. Mater..

[23] Zhang S. (2003). Fabrication of novel biomaterials through molecular self-assembly. Nat. Biotechnol..

[24] Boal A.K., Ilhan F., DeRouchey J.E., Thurn-Albrecht T., Russell T.P., Rotello V.M. (2000). Self-assembly of nanoparticles into structured spherical and network aggregates. Nature.

[25] Cui W., Li J., Decher G. (2016). Self-Assembled Smart Nanocarriers for Targeted Drug Delivery. Adv. Mater..

[26] Whitesides G.M., Mathias J.P., Seto C.T. (1991). Molecular Self-Assembly and Nanochemistry: A Chemical Strategy for the Synthesis of Nanostructures. Science.

[27] Kolay S., Bain D., Maity S., Devi A., Patra A., Antoine R. (2022). Self-Assembled Metal Nanoclusters: Driving Forces and Structural Correlation with Optical Properties. Nanomaterials.

[28] Koutsopoulos S. (2012). Molecular fabrications of smart nanobiomaterials and applications in personalized medicine. Adv. Drug Deliv. Rev..

[29] Michiue H., Kitamatsu M., Fukunaga A., Tsuboi N., Fujimura A., Matsushita H., Igawa K., Kasai T., Kondo N., Matsui H. (2021). Self-assembling A6K peptide nanotubes as a mercaptoundecahydrododecaborate (BSH) delivery system for boron neutron capture therapy (BNCT). J. Control. Release.

[30] Yoneoka S., Park K.C., Nakagawa Y., Ebara M., Tsukahara T. (2019). Synthesis and evaluation of thermoresponsive boron-containing poly(*N*-isopropylacrylamide) diblock copolymers for self-assembling nanomicellar boron carriers. Polymers.

[31] Qiu S., Hou Y., Xing W., Ma C., Zhou X., Liu L., Kan Y., Yuen R.K.K., Hu Y. (2018). Self-assembled supermolecular aggregate supported on boron nitride nanoplatelets for flame retardant and friction application. Chem. Eng. J..

[32] Vinothini K., Rajendran N.K., Ramu A., Elumalai N., Rajan M. (2019). Folate receptor targeted delivery of paclitaxel to breast cancer cells via folic acid conjugated graphene oxide grafted methyl acrylate nanocarrier. Biomed. Pharmacother..

[33] Zhang H., Feng S., Yan T., Huang D., Zhi C., Nakanishi H., Gao X.-D. (2016). Folate-conjugated boron nitride nanospheres for targeted delivery of anticancer drug. Int. J. Nanomed..

[34] Nasrollahzadeh M., Sajadi S.M., Sajjadi M., Issaabadi Z. (2019). Applications of Nanotechnology in Daily Life. Interface Science and Technology.

[35] Drexler K.E. (1988). The Coming Era of Nanotechnology. The Materials Revolution. Superconductors, New Materials and the Japanese Challenge.

[36] Buzea C., Pacheco I.I., Robbie K. (2007). Nanomaterials and nanoparticles: Sources and toxicity. Biointerphases.

[37] Silva G.A. (2004). Introduction to nanotechnology and its applications to medicine. Surg. Neurol..

[38] Caruthers S.D., Wickline S.A., Lanza G.M. (2007). Nanotechnological applications in medicine. Curr. Opin. Biotechnol..

[39] Singhal R., Echeverria E., McIlroy D.N., Singh R.N. (2021). Synthesis of hexagonal boron nitride films on silicon and sapphire substrates by low-pressure chemical vapor deposition. Thin Solid Films.

[40] Chen X., Tan C., Liu X., Luan K., Guan Y., Liu X., Zhao J., Hou L., Gao Y., Chen Z. (2021). Growth of hexagonal boron nitride films on silicon substrates by low-pressure chemical vapor deposition. J. Mater. Sci. Mater. Electron..

[41] Hayat A., Sohail M., Hamdy M.S., Taha T.A., AlSalem H.S., Alenad A.M., Amin M.A., Shah R., Palamanit A., Khan J. (2022). Fabrication, characteristics, and applications of boron nitride and their composite nanomaterials. Surf. Interfaces.

[42] Yang H., Li J., Gu S., Wu Z., Luo L., Chen Y. (2022). Fabrication of hexagonal boron carbonitride nanoplates using for in vitro photodynamic therapy and chemo therapy. Colloids Surf. B Biointerfaces.

[43] Ciofani G., Raffa V., Menciassi A., Cuschieri A. (2009). Folate Functionalized Boron Nitride Nanotubes and their Selective Uptake by Glioblastoma Multiforme Cells: Implications for their Use as Boron Carriers in Clinical Boron Neutron Capture Therapy. Nanoscale Res. Lett..

[44] Dehghan Banadaki M., Aghaie M., Aghaie H. (2021). Folic acid functionalized boron nitride oxide as targeted drug delivery system for fludarabine and cytarabine anticancer drugs: A DFT study. J. Mol. Liq..

[45] Guo W., Lee R.J. (2001). Efficient gene delivery via non-covalent complexes of folic acid and polyethylenimine. J. Control. Release.

[46] Feng S., Zhang H., Zhi C., Gao X.D., Nakanishi H. (2018). pH-responsive charge-reversal polymer-functionalized boron nitride nanospheres for intracellular doxorubicin delivery. Int. J. Nanomed..

[47] Teo P.Y., Yang C., Whilding L.M., Parente-Pereira A.C., Maher J., George A.J.T., Hedrick J.L., Yang Y.Y., Ghaem-Maghami S. (2015). Ovarian Cancer Immunotherapy Using PD-L1 siRNA Targeted Delivery from Folic Acid-Functionalized Polyethylenimine: Strategies to Enhance T Cell Killing. Adv. Healthc. Mater..

[48] Zhang G., Zhang M., He J., Ni P. (2013). Synthesis and characterization of a new multifunctional polymeric prodrug paclitaxel–polyphosphoester–folic acid for targeted drug delivery. Polym. Chem..

[49] Talesa V.N. (2001). Acetylcholinesterase in Alzheimer’s disease. Mech. Ageing Dev..

[50] Lazarevic-Pasti T., Leskovac A., Momic T., Petrovic S., Vasic V. (2017). Modulators of Acetylcholinesterase Activity: From Alzheimer’s Disease to Anti-Cancer Drugs. Curr. Med. Chem..

[51] Saxena M., Dubey R. (2019). Target Enzyme in Alzheimer’s Disease: Acetylcholinesterase Inhibitors. Curr. Top. Med. Chem..

[52] Marucci G., Buccioni M., Ben D.D., Lambertucci C., Volpini R., Amenta F. (2021). Efficacy of acetylcholinesterase inhibitors in Alzheimer’s disease. Neuropharmacology.

[53] Subramaniam S.R., Chesselet M.F. (2013). Mitochondrial dysfunction and oxidative stress in Parkinson’s disease. Prog. Neurobiol..

[54] Cassidy L., Fernandez F., Johnson J.B., Naiker M., Owoola A.G., Broszczak D.A. (2020). Oxidative stress in alzheimer’s disease: A review on emergent natural polyphenolic therapeutics. Complement. Ther. Med..

[55] Türkez H., Arslan M.E., Sönmez E., Açikyildiz M., Tatar A., Geyikoğlu F. (2019). Synthesis, characterization and cytotoxicity of boron nitride nanoparticles: Emphasis on toxicogenomics. Cytotechnology.

[56] Turkez H., Arslan M.E., Yilmaz A., Doru F., Caglar O., Arslan E., Tatar A., Hacımuftuoglu A., Abd El-Aty A.M., Mardinoglu A. (2021). In vitro transcriptome response to propolis in differentiated SH-SY5Y neurons. J. Food Biochem..

